# Bentall procedure for retrograde type A dissection after endovascular repair in type A aortic dissection: A case report

**DOI:** 10.1097/MD.0000000000029615

**Published:** 2022-08-05

**Authors:** Li Ma, Long Liu, Sheng Yan, Jun Yan

**Affiliations:** a Department of Anesthesiology, Chinese Academy of Medical Sciences and Peking Union Medical College Hospital, Beijing, China; b Department of Cardiovascular Surgery, Taiyuan Central Hospital of Shanxi Medical University, Taiyuan, China.

**Keywords:** aortic dissection, Bentall procedure, TEVAR

## Abstract

**Rationale:**

The management of retrograde type A dissection (RTAD) after thoracic endovascular aortic repair (TEVAR) for type A aortic dissection (TAAD) has rarely been reported. We report the management of RTAD after TEVAR with in situ fenestration for TAAD.

**Patient concerns:**

A 59-year-old man with TAAD had undergone TEVAR with in situ fenestration 4 months prior to presenting to our emergency room complaining of acute chest and back pain. Computed tomography angiography showed RTAD starting from the proximal endograft and extending to the aortic root.

**Diagnosis:**

The patient was diagnosed with RTAD.

**Interventions:**

We performed only the Bentall procedure, and the patient did not require total arch replacement. We removed the bare spring of the proximal endograft and anastomosed the prosthetic graft with the endograft and the native ascending aortic wall.

**Outcomes:**

The postoperative course was uneventful, and the patient remained asymptomatic for 3 years after surgery. Computed tomography angiography at the 3-year follow-up showed no perivalvular or anastomotic leakage.

**Lessons:**

RTAD after TEVAR for TAAD was safely and effectively treated by anastomosing the prosthetic graft with the endograft and the native ascending aortic wall instead of total arch replacement.

## 1. Introduction

Acute type A aortic dissection (TAAD) is a life-threatening disease with a 24-hour mortality rate of 50% while waiting for surgical treatment, with open surgery being the gold standard treatment for TAAD.^[1,2]^ However, thoracic endovascular aortic repair (TEVAR) has recently been used as an alternative treatment for type A aortic disease in patients who are poor candidates for surgical repair.^[3,4]^ Retrograde type A dissection (RTAD) is a life-threatening complication after TEVAR.^[3]^ Here, we report a case of RTAD after TEVAR with an in situ-fenestrated endograft for TAAD that was successfully treated using the Bentall procedure and did not require total arch replacement.

## 2. Case Report

**Figure 1.**
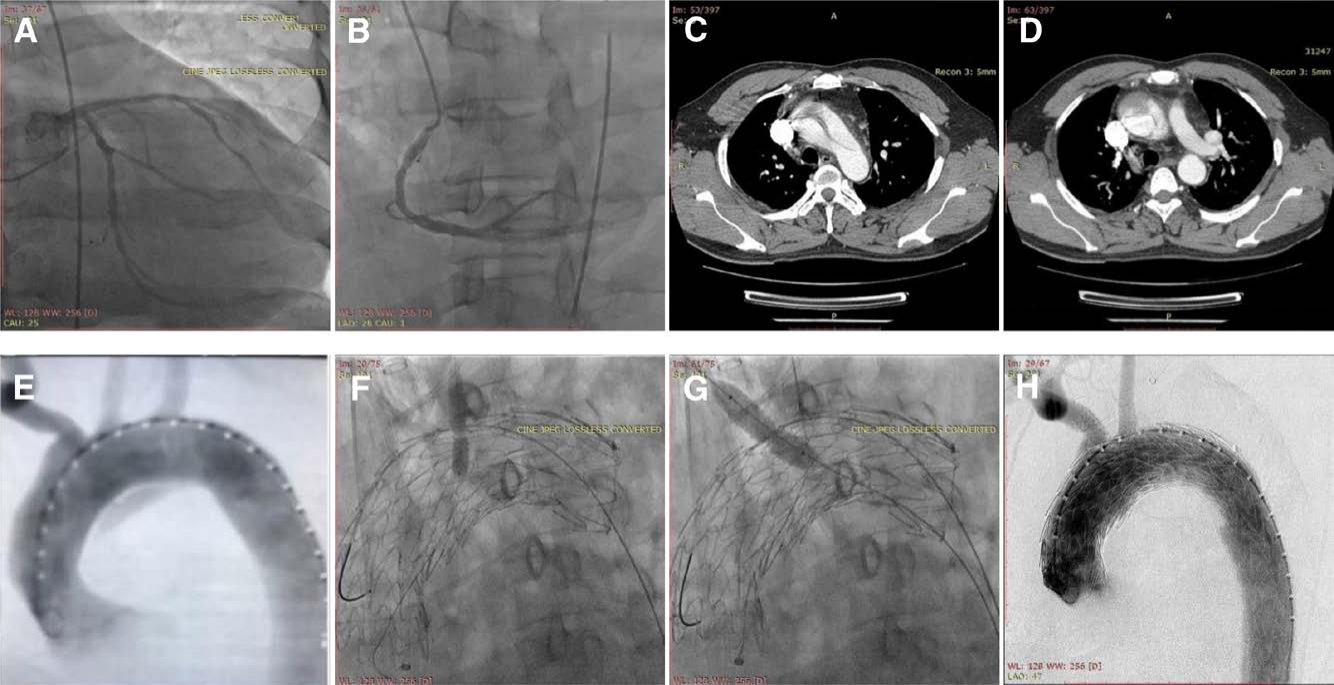
The perioperative images of the thoracic endovascular aortic repair. (A) Coronary angiography showing that 30%–40% of stenosis was in the proximal-middle segment of the left anterior descending artery. (B) Coronary angiography showing that the right coronary artery was not obviously stenotic. (C) CTA showing that the intimal tear started from the distal ascending aorta at the inner curve of the ascending aorta. (D) The distal tear extended to the left subclavian artery. (E) The angiography showing the location of the dissection. (F) The balloon placed for predilatation via the left common carotid artery in a retrograde way. (G) The balloon placed for predilatation via the right common carotid artery in a retrograde way. (H) The aortogram showing the left and right common carotid arteries were patent without any endoleak, but the left subclavian artery failed to reconstruct. CTA = computed tomography angiography.

A 59-year-old man with a medical history of hypertension without medical management presented to another hospital emergency room with acute-onset chest and back pain 4 months prior to presenting to our hospital. He was given a loading dose of clopidogrel (300 mg) and aspirin (300 mg) due to suspicion of acute myocardial infarction based on both electrocardiography and myocardial enzyme test results, but emergency coronary angiography (**Fig. 1A**, B) showed 30% to 40% stenosis in the proximal-middle segment of the left anterior descending artery. Subsequently, computed tomography angiography (CTA; **Fig. 1C**, D) and aortic angiography (**Fig. 1E**) showed an acute TAAD in which there was an intimal tear originating from the inner curve of the ascending aorta extending distally to the left subclavian artery. Finally, TEVAR with in situ fenestration was performed. A thoracic endograft (Gore, 40–40–150 mm, C-TAG) was inserted and deployed to the ascending aorta. The thoracic endograft was punctured using a liver biopsy needle (Cook; length, 15 cm; gauge, 22) via the left common carotid artery in a retrograde manner. A 0.018-inch wire was passed through to establish a working path. A balloon (Boston Scientific, 4–40, 8–40 mm, Mustang) was then placed for predilatation (**Fig. 1F**). An endograft (Gore, 9–50 mm, Viabahn) was next deployed across the fenestration. The same approach was applied to create a fenestration for the right common carotid artery. A 0.018-inch wire was passed, and a balloon (Boston Scientific, 4–30, 6–100 mm, Mustang) was placed for predilatation (**Fig. 1G**). An endograft (Gore, 8–50 mm, Viabahn) was deployed across the fenestration. A RUPS-100 needle (Cook) failed to create a fenestration and allow reconstruction of the left subclavian artery. A second distal thoracic endograft (Gore, 37–37–150 mm, C-TAG) was deployed. Angiography showed that the left and right common carotid arteries were patent, without any endoleakage (**Fig. 1H**).

**Figure 2.**
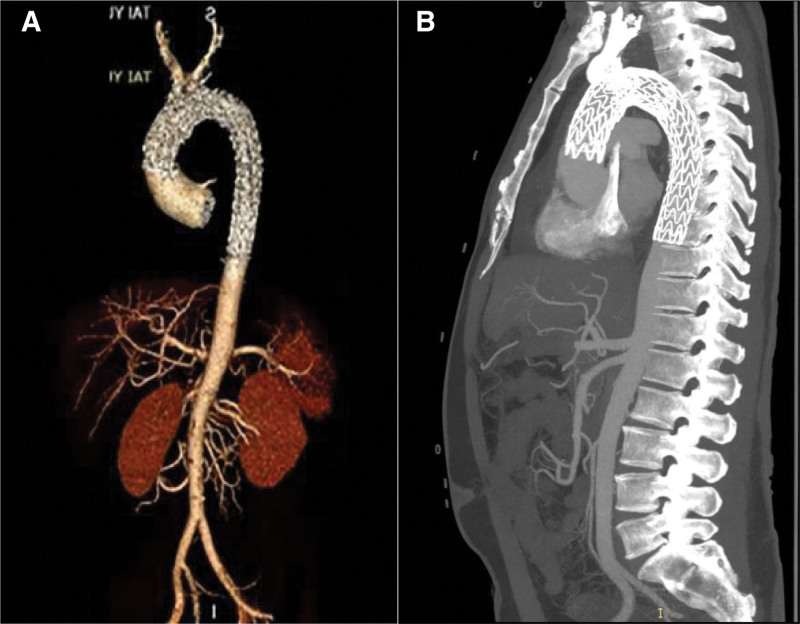
The images of the 3-mo follow-up. CTA showing that the aortic remodeling was good, and there was no stent graft-induced redissection. CTA = computed tomography angiography.

**Figure 3.**
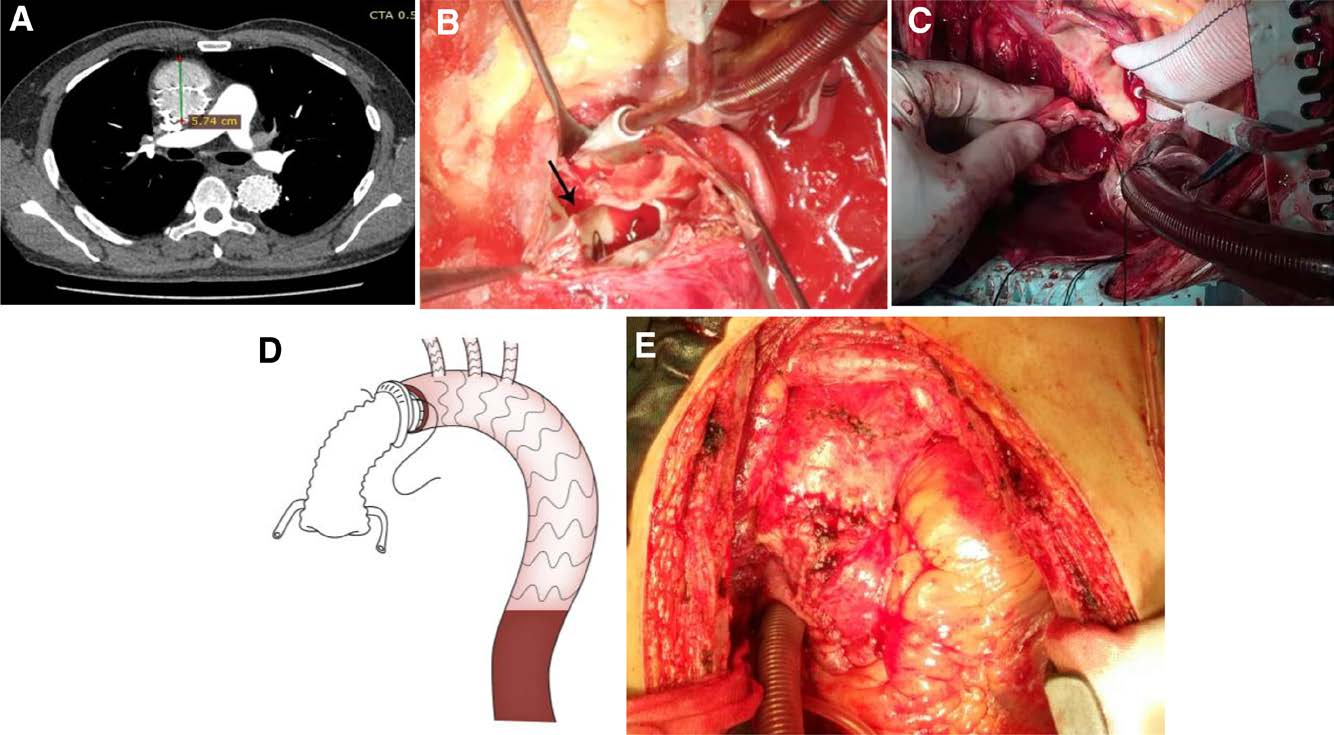
The perioperative images of the second operation. (A) CTA showing a new retrograding dissection starting from the proximal endograft and extending proximally to the aortic root with a maximum diameter of 57.4 mm. (B) Intraoperative image after incision of the ascending aorta. The arrow indicates the entry tear located in the proximal bare spring of the endograft. (C) The steel wires of the bare spring were cut to create an appropriate distal plane. (D) The graft inversion technique of distal anastomosis. (F) The aorta and grafts were wrapped and sutured with pericardium. CTA = computed tomography angiography.

The blood pressure and heart rate were well controlled after the surgery. At the 3-month follow-up, the patient did not complain of any discomfort, and CTA showed good aortic remodeling without graft-induced redispersion (**Fig. 2**). However, 4 months later, he presented to our emergency room and complained of acute chest and back pain. Another CTA examination showed a new RTAD starting from the proximal endograft and extending proximally to the aortic root, with a maximum diameter of 57.4 mm (**Fig. 3A**). Echocardiography showed severe aortic root dilation with severe aortic regurgitation (aortic sinus, 43.2 mm; ascending aorta, 55.9 mm) but good ventricular function.

We performed emergency surgery through a median sternotomy under general anesthesia. After systemic heparinization, cardiopulmonary bypass was established by arterial cannulation through the left common femoral artery and placement of a dual-stage venous cannula through the right atrium. A left heart drainage tube was inserted into the right superior pulmonary vein, and cardiopulmonary bypass was initiated. The ascending aorta was clamped at the end and incised, the new intimal flap was placed at the tip of the proximal bare spring, and the dissection extended into the aortic sinus (**Fig. 3B**). Protection of the myocardium was achieved by the intermittent infusion of a cardioplegia solution every 30 minutes. When the aortic valves were exposed, the aortic leaflets were resected. Then, a size-matched prosthetic valve conduit was selected and attached to the annulus with pledged-reinforced horizontal mattress sutures of 2–0 braided polyester. The coronary artery was subsequently anastomosed with the graft using continuous sutures of 5–0 polypropylene. The steel wires of the bare spring were cut to create an appropriate distal plane (**Fig. 3C**). The prosthetic graft was anastomosed with the previous endograft and the native ascending aortic wall (**Fig. 3D**), which was reinforced using a felt strip. The aorta was wrapped and sutured to the pericardium (**Fig. 3E**). The patient was then weaned off cardiopulmonary bypass, and the chest was closed.

**Figure 4.**
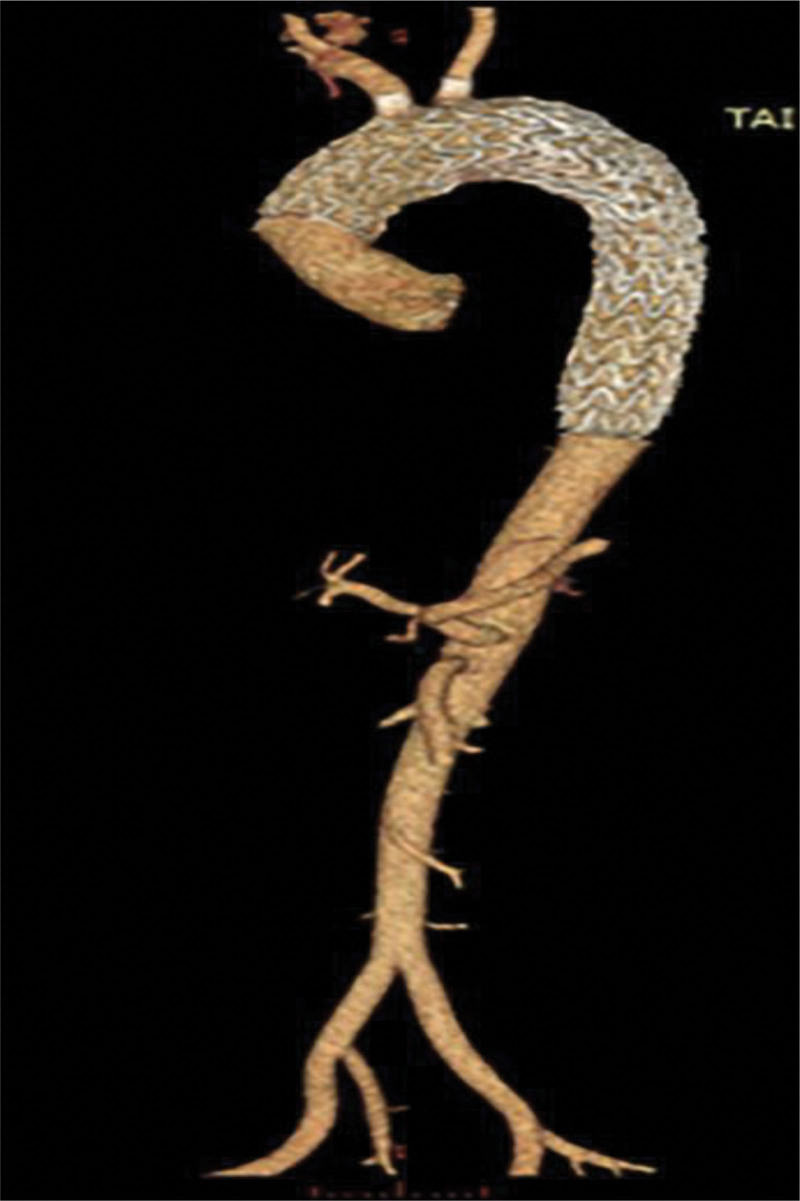
The 3-yr follow-up CTA. CTA shows that there was no perivalvular leakage in the aortic root or pseudoaneurysm in the anastomosis of the ascending aorta. CTA = computed tomography angiography.

The patient’s postoperative course was uneventful, and he was discharged from the hospital on the 8th day after surgery. He did not complain of any discomfort during the follow-up period. CTA at the 3-year follow-up showed no perivalvular or anastomotic leakage or pseudoaneurysms (**Fig. 4**). This study was conducted with the approval of the ethics committee and the patient or authorized person.

## 3. Discussion

In recent years, TEVAR has become the first-line treatment for complicated type B aortic dissection (TBAD)^[2]^ and has also been used in treating type A aortic disease in patients who are unsuitable candidates for surgical repair.^[3,5]^ However, we cannot ignore the fact that RTAD is a rare but lethal complication after TEVAR,^[3,4]^ and RTAD is more common after TEVAR for TAAD than for TBAD. Lu et al^[4]^ reported that the incidence of RTAD in TAAD was nearly 15%, while it was 1.3% to 8% in TBAD.^[6,7]^ Several factors may contribute to these findings. First, the ascending aorta is subjected to high velocities and consequent shear stresses under pulsatile movements during the cardiac and respiratory cycles. This can lead to proximal leakage and rupture from repeated friction between the endograft and aortic wall.^[4,6]^ Second, an increased radial force due to the curvature of the aortic arch and stent oversizing may injure the aortic wall. Third, blood pressure and heart rate instability can also increase the incidence of TAAD compared to TBAD; this is why TEVAR cannot be regularly used in the treatment of acute TAAD.

Regarding the surgical management of patients with RTAD after TEVAR for TBAD, the technique mainly involves total or hemiarch replacement with or without the frozen elephant trunk technique, which depends on the aortic wall. That is, if the aortic wall is fragile and injured, the frozen elephant trunk stent is inserted into the descending aorta to strengthen it.^[7–9]^ In the management of existing endografts, the appearance of a new tear in the fragile aortic wall during endograft removal must be considered, so in most cases, these endografts are left in place, and the frozen elephant trunk technique is omitted; however, the bare spring of the proximal endograft would be removed to create an appropriate distal plane.^[6,7,10]^

However, the surgical management of RTAD after TEVAR for TAAD has seldom been reported.^[11–13]^ In contrast to RTAD in TBAD, RTAD in TAAD requires the resolution of aortic arch and endograft lesions if total arch replacement is performed. In the present case, the previously implanted branched stents and endografts in the arch needed to be removed when the patient was under deep hypothermic circulatory arrest and had cerebral protection. The processes were highly challenging and complicated because these stents were anchored to the aortic wall. As described above, endografts were also placed for the management of RTAD after TEVAR for type A aortic disease. Kasahara et al^[12]^ removed the proximal endograft and anastomosed the prosthetic graft with the distal ascending aorta instead of the endograft after TEVAR with the chimney graft technique for an aortic arch aneurysm in TAAD. Maximilian et al^[14]^ also performed 2 related surgeries in which the prosthetic graft was anastomosed with the end of the endograft in RTAD following complete supra-aortic debranching and stent grafting of the transverse arch. In this patient, who was in an emergent state, it was feasible to leave the endografts and just remove the bare spring, while the prosthetic graft was anastomosed with the endograft and the native ascending aortic wall. There are several advantages to this technique. One is that it simplifies the surgical process and shortens the operation by avoiding aortic arch replacement. Another is that deep hypothermic circulatory arrest and selective cerebral perfusion are not needed, and the risk of cerebral complications is reduced. The patient showed no perivalvular leakage in the aortic root or pseudoaneurysms at the site of anastomosis of the ascending aorta at the 3-year follow-up in this study.

## 4. Conclusion

We report the first case of RTAD after TEVAR with an in situ-fenestrated endograft for TAAD that was successfully repaired with the Bentall procedure and anastomosis of the distal prosthetic graft with the endograft and ascending aortic wall without total arch replacement. Further studies are necessary to identify the optimal surgical process for RTAD after TEVAR for TAAD.

## Author contributions

Conceptualization: Jun Yan

Data curation: Li Ma, Long Liu, Sheng Yan, Jun Yan

Formal analysis: Li Ma, Sheng Yan, Jun Yan

Investigation: Li Ma, Long Liu, Jun Yan

Supervision: Sheng Yan, Jun Yan

Writing – original draft: Li Ma

Writing – review & editing: Sheng Yan, Jun Yan
